# Memory type Max-EWMA control chart for the Weibull process under the Bayesian theory

**DOI:** 10.1038/s41598-024-52109-0

**Published:** 2024-02-07

**Authors:** Muhammad Noor-ul-Amin, Imad Khan, Javed Iqbal, Zahid Rasheed, Emad A. A. Ismail, Bakhtyar Ahmad

**Affiliations:** 1 COMSATS University Islamabad, Lahore Campus, Lahore, Pakistan; 2 Abdul Wali Khan University Mardan, Mardan, Pakistan; 3 School of Mathematics and Statistics, Xi’an Jiaotong University, Xi’an, Shaanxi China; 4 Department of Quantitative Analysis, College of Business Administration, King Saud University, P.O. Box 71115, 11587 Riyadh, Saudi Arabia; 5 Higher Education Department Afghanistan, Kabul, Afghanistan

**Keywords:** Engineering, Scientific data, Statistics

## Abstract

The simultaneous monitoring of both process mean and dispersion, particularly in normal processes, has garnered significant attention within the field. In this article, we present a new Bayesian Max-EWMA control chart that is intended to track a non-normal process mean and dispersion simultaneously. This is accomplished through the utilization of the inverse response function, especially in cases where the procedure follows a Weibull distribution. We used the average run length (ARL) and the standard deviation of run length (SDRL) to assess the efficacy of our suggested control chart. Next, we contrast our suggested control chart's performance with an already-existing Max-EWMA control chart. Our results show that compared to the control chart under consideration, the proposed control chart exhibits a higher degree of sensitivity. Finally, we present a useful case study centered around the hard-bake process in the semiconductor manufacturing sector to demonstrate the performance of our Bayesian Max-EWMA control chart under different Loss Functions (LFs) for a Weibull process. The case study highlights how flexible the chart is to various situations. Our results offer strong proof of the outstanding ability of the Bayesian Max-EWMA control chart to quickly identify out-of-control signals during the hard-bake procedure. This in turn significantly contributes to the enhancement of process monitoring and quality control.

## Introduction

In statistical quality control, distributions such as the Weibull are commonly used for the analysis of reliability data, which frequently involves failure times or life-test experiments. There are two primary categories of variations in processes that follow these distributions: assignable cause and common cause. Despite common cause variation occurring naturally and arbitrarily within the process, it is still considered under control. Assignable cause variation, on the other hand, denotes an out-of-control departure from the typical process state. Walter A. Shewhart^1^ pioneered control charts (CCs) and initially concentrating on using current sample data to identify changes in production processes, serve as tools to detect and address such deviations. Memory-type control charts like cumulative sum (CUSUM) and exponentially weighted moving average (EWMA), introduced by Page^2^ and Roberts^3^, have significantly improved sensitivity by incorporating both current and historical data. This advancement has been crucial in identifying subtle to moderate shifts in process parameters. Industries relying on precision, such as chemicals and manufacturing, extensively use CUSUM and EWMA control charts to promptly detect variations and maintain high product quality standards. Gen^4^ evaluated control-charting methods for monitoring process mean and variance concurrently, revealing limitations in some individual schemes. They proposed a combined scheme using two-sided EWMA charts for mean and variance, effectively identifying out-of-control situations. This study not only offers methods to estimate average run length (ARL) and run-length distribution percentages for this combined EWMA scheme but also provides a design procedure. Chen et al.^5^ introduced a novel EWMA control chart that integrates monitoring of process mean and variability into a single chart. This innovation enables the detection of both increases and decreases in mean and/or variability. BC Khoo et al.^6^ introduces the Max-DEWMA chart, an extension of the Max-EWMA, utilizing statistics derived from two DEWMA statistics for mean and variance, demonstrating superior performance in detecting small to moderate shifts in mean and/or variance. Huei Sheu et al.^7^ studied the Max-GWMA CC, detecting mean and/or variability changes simultaneously, outperforming the Max-EWMA chart in sensitivity through comprehensive simulations and diagnostic assessments. Sheu et al.^7^ developed the Max-GWMA CC, excelling in detecting changes in mean and variability. Simulations demonstrate its heightened sensitivity compared to the Max-EWMA chart, rendering it a valuable tool for monitoring process variations. Sanusi et al.^8^ presents four EWMA schemes for joint monitoring of Gaussian process mean and variance, comparing 'max' and 'distance' combining functions, revealing the superiority of distance-type schemes in detecting shifts with faster identification from industrial datasets. Arif et al.^9^ introduced a novel CC combining EWMA and generalized likelihood ratio test for simultaneous mean and dispersion monitoring using double RSS. Comparative simulations showcase its superior performance in simultaneous shift detection compared to RSS and PRSS charts, with real data applications demonstrating practical utility. Noor-ul-Amin et al.^10^ introduced a Max-EWMA CC for joint mean and dispersion monitoring in Weibull-distributed processes using the inverse response function. Their evaluation, using ARL and SDRL, demonstrated higher sensitivity compared to an existing Max-EWMA CC, illustrated through practical examples. Yang^11^ introduced an enhanced Qpm MQCAC method, aiming for efficient product quality optimization by addressing excessive or inadequate quality issues. This approach monitors process mean and standard deviation shifts, identifies influential factors, and minimizes resource consumption according to GM objectives. A practical demonstration using a steering knuckle pin example is provided, along with insights into potential future research directions. Chatterjee et al.^12^ extended the Max-EWMA CC to develop a single Max-GWMA CC for concurrent process mean and variability monitoring. Comparative analysis with Max-EWMA and Max-DEWMA charts revealed the efficiency of the Max-GWMA chart in detecting small shifts in both parameters, demonstrated through practical implementations using real and simulated datasets. Saemian et al.^13^ addressed concurrent process mean and variability monitoring in SPM with the Max-HEWMAMS CC, mitigating measurement imprecision due to gauge inaccuracies. Employing multiple measurements to reduce measurement errors' impact on chart detection, they highlighted benefits through various out-of-control scenarios, showcasing the negative effect of gauge imprecision using real data. Abbas et al.^14^ presented Bayesian CUSUM CCs for statistical process control profile monitoring, proving their superiority over rival techniques in an extensive comparative analysis. This highlighted the benefits of Bayesian methods and demonstrated their superiority with case studies and simulations that needed in-depth process parameter data. Erto et al.^15^ carried out a simulation study assessing semi-empirical Bayesian CCs for tracking Weibull distribution data. They utilized Weibull contour plots and reference data to provide real-world examples to illustrate the effects of changing Weibull parameters through Monte Carlo analysis. Erto et al.^16^ reported a new Bayesian CC technique that compares two processes by monitoring the ratio of the independent Weibull-distributed quality characteristics' percentiles. This graph demonstrated its performance using extensive simulations and real-world applications in the wood industry, taking into account variables such as shift magnitude, training data quality, and prior information quality. A Bayesian modified EWMA CC with four LFs and a conjugate prior was introduced by Aslam and Anwar^17^, who found that it was very effective at detecting small to moderate process shifts. Validation included monitoring the mechanical industry’s reaming process and assessing sports industry golf ball performance. Lin et al.^18^ introduced a Bayesian procedure constructing an EWMA CC for monitoring the variance of a distribution-free service process, capable of handling non-normal and time-varying distributions. Demonstrations with bank service time displayed its efficacy in quickly detecting variance shifts. Noor-ul-Amin and Noor^19^ proposed an AEWMA CC integrating Shewhart and EWMA approaches to detect various shifts in process mean. Bayesian theory with LFs and informative priors was employed, validated through Monte Carlo simulations and real-data examples. Yazdi et al.^20^ developed Bayesian CCs for monitoring multivariate linear profiles using regression models, outperforming non-Bayesian counterparts based on ARL criteria. They introduced a historical data-driven informative prior method, demonstrating applicability through extensive simulations. Khan et al.^21^ introduced a novel Bayesian AEWMA CC incorporating RSS designs, SELF, LLF, and an informative prior. Monte Carlo simulations validated its sensitivity in detecting mean shifts, exemplified in a semiconductor fabrication process. Furthermore, Khan et al.^22^ introduced a Bayesian HEWMA CC using RSS schemes with informative priors and various LFs, showing enhanced sensitivity in detecting out-of-control signals. The article uniquely focuses on Bayesian methods for joint monitoring of mean and variance, specifically for lifetime data. It introduces a Bayesian Max-EWMA CC for simultaneous monitoring of mean and variance in Weibull processes, evaluated through Monte Carlo simulations.. The article is structured with sections dedicated to Bayesian theory and various LFs in “Bayesian approach”, the proposed Bayesian Max-EWMA CC method in “Proposed Bayesian Max-EWMA CC for joint monitoring of the Weibull distribution”, comprehensive discussions in “Results and discussion”, “Main findings” contain the key findings, practical applications using real-life data in “Real data application”, and concluding remarks in “Conclusion”.

## Bayesian approach

The Bayesian approach uses probability theory to model and analyze uncertainty, treating model parameters as random variables with associated probability distributions. It incorporates prior beliefs and updates them with observed data, resulting in posterior probability distributions. The key steps include defining prior distributions, likelihood functions for data generation, combining priors and likelihoods for posteriors, and Bayesian inference. This reiterative method provides a coherent outline, is robust to outliers and small samples, is flexible, integrates prior knowledge, allows for continuous improvement with new data, and quantifies uncertainty. Although it can be computationally exhaustive for complex models and requires prior assumptions, it is widely used in areas such as scientific research, data science, and machine learning. Consider a random variable denoted as *V*, representing lifetimes, and assume that it follows a Weibull distribution characterized by the shape parameter ($\lambda$) and scale parameter ($\alpha$), both of which are greater than zero (*a* > 0,* k* > 0). The probability density function (pdf) and cumulative distribution function (cdf) are mathematically described as follows:

1 $$f\left( {v,\alpha ,\lambda } \right) = \frac{{\lambda v^{{\left( {\lambda - 1} \right)}} }}{{\alpha^{\lambda } }}\exp \left( { - \left( {\frac{v}{\alpha }} \right)^{\lambda } } \right);\quad v > 0,$$

2 $$F\left( v \right) = \Pr \left( {V \le v} \right) = 1 - \exp \left( { - \left( {\frac{v}{\alpha }} \right)^{\lambda } } \right);\quad v = 0.$$

### Squared error loss function

To assess the discrepancy between expected and actual values, the squared error loss function—also called mean square error or MSE—is essential in Bayesian methodology. It is essential to both Bayesian inference and decision theory. Predictions or parameter estimates are represented as probability distributions in Bayesian modeling. The squared error loss measures the cost of differences by squaring them mathematically, which penalizes larger discrepancies more severely. The primary goal of Bayesian practice is to minimize the expected squared error loss by averaging it under the posterior distribution, resulting in point estimates or predictive distributions. This loss function is widely used in Bayesian applications, especially for continuous variables, and is closely related to the mean squared error. Ultimately, it helps assess the quality of predictions and parameter estimates by considering both estimate uncertainty and their proximity to actual values. The SELF is endorsed by Gauss ^23^, incorporating both the variable of interest, denoted as *X*, and the estimator $\hat{\theta }$ used for estimating an unknown population parameter $\theta$, denoted as theta. Its mathematical expression is as follows:

3 $$L\left( {\theta ,\hat{\theta }} \right) = \left( {\theta - \hat{\theta }} \right)^{2}$$

And the Bayes estimator using SELF is mathematized.

4 $$\hat{\theta }_{{\left( {SELF} \right)}} = E_{\theta /x} \left( \theta \right).$$

### Linex loss function

The Linex loss function employed in Bayesian analysis measures the cost of prediction errors by balancing linear and exponential components. It assesses the difference between predicted and true values and is valuable in various Bayesian applications, allowing flexibility in quantifying asymmetric losses and adapting to scenarios with varying consequences of overestimation and underestimation errors. Varian ^24^ proposed LLF to mitigate risks in Bayes estimation. The LLF is mathematically described

5 $$L\left( {\theta ,\hat{\theta }} \right) = \left( {e^{{c\left( {\theta - \hat{\theta }} \right)}} - c\left( {\theta - \hat{\theta }} \right) - 1} \right)$$

Under LLF, the Bayesian estimator $\hat{\theta }$ is mathematizied as

6 $$\hat{\theta }_{{\left( {LLF} \right)}} = - \frac{1}{c}InE_{\theta /x} \left( {e^{ - c\theta } } \right).$$

## Proposed Bayesian Max-EWMA CC for joint monitoring of the Weibull distribution

We have a series of random samples, denoted as *V*_*i1*_*, V*_*i2*_*, …, V*_*in*_, drawn from a Weibull distribution denoted as $W(\alpha ,\lambda )$ at different time points, i.e.,* i* = 1, 2, 3. Typically, the Weibull distribution’s parameters ($\alpha$ and $\lambda$) are not known in advance and need to be estimated using available historical data. To estimate these parameters, an appropriate method is employed, usually with the consideration that the process is under control. Letus denote the estimated scale and shape parameters as $\alpha_{0}$ and $\lambda_{0}$, respectively. These parameter estimates are derived by leveraging a relationship between the Weibull and standard normal distributions, as given by Faraz et al. ^25^ in Eq. (7) as follows:

7 $$\phi \left( {z,\alpha ,\lambda } \right) = f\left( {W^{ - 1} \left( {z,\alpha ,\lambda } \right)} \right)\frac{dt}{{dz}}$$

where the mean and variance are given by

8 $$\mu_{z} = E\left( {Z,\alpha ,\lambda } \right) = \int z \phi \left( {z,\alpha ,\lambda } \right)dz$$

And

9 $$\delta_{z}^{2} = Var\left( {Z,\alpha ,\lambda } \right) = \int {\left( {z - \mu } \right)^{2} } \phi \left( {z,\alpha ,\lambda } \right)dz.$$

Equations (8) and (9) provides insights into how shifts in the parameters of a Weibull distribution influence the mean and variance of a random variable following a standard normal distribution. Essentially, they quantify the impact of changing Weibull distribution parameters on the characteristics of the standard normal distribution.

Consider random samples: $z_{i1} = W\left( {V_{i1} ,\alpha_{0} ,\lambda_{0} } \right)$, $z_{i2} = W_{N} \left( {V_{i2} ,\alpha_{0} ,\lambda_{0} } \right)$, …, $z_{in} = W_{N} \left( {V_{in} ,\alpha_{0} ,\lambda_{0} } \right)$. Each of these samples has a size of *n* and is transformed from a Weibull to a normal distribution. After this transformation, we introduce the Max-EWMA Control Chart, which leverages Bayesian theory to concurrently monitor the mean and variance of a normally distributed process.

10 $$f\left( {z_{t} :\theta ,\sigma^{2} } \right) = \frac{1}{{\sqrt {2\pi \sigma^{2} } }}\exp \left( { - \tfrac{1}{{2\sigma^{2} }}\left( {z_{t} - \theta } \right)^{2} } \right).$$

In a Bayesian framework, when both the likelihood function and prior distribution are normally distributed, the resulting posterior distribution also follows a normal distribution characterized by a mean (θ) and variance (σ). The pdf is as follows:

11 $$P\left( {\theta /z} \right) = \frac{1}{{\sqrt {2\pi } \sqrt {\frac{{\delta^{2} \delta_{0}^{2} }}{{\delta^{2} + n\delta_{0}^{2} }}} }}\exp \left[ { - \frac{1}{2}\left( {\frac{{\theta - \sum\limits_{i = 1}^{n} {\frac{{z_{i} \delta_{0}^{2} + \theta_{0} \delta_{0}^{2} }}{{\delta^{2} + n\delta_{0}^{2} }}} }}{{\sqrt {\frac{{\delta^{2} \delta_{0}^{2} }}{{\delta^{2} + n\delta_{0}^{2} }}} }}} \right)^{2} } \right]$$

where $\theta_{n} = \frac{{n\overline{z}\delta_{0}^{2} + \delta^{2} \theta_{0} }}{{\delta^{2} + n\delta_{0}^{2} }}$ and $\delta_{n}^{2} = \frac{{\delta^{2} \delta_{0}^{2} }}{{\delta^{2} + n\delta_{0}^{2} }}$ respectively.

To create a Max-EWMA chart using Bayesian methodology, we begin by selecting a sample of *n* values for a quality characteristic *Z* from the production process. Subsequently, we compute transformed statistics under SELF for both the mean and variance as follows

12 $$U_{t} = \frac{{\hat{\theta }_{(SELF)} - \theta }}{{{\raise0.7ex\hbox{$\delta $} \!\mathord{\left/ {\vphantom {\delta {\sqrt n }}}\right.\kern-0pt} \!\lower0.7ex\hbox{${\sqrt n }$}}}}$$

and

13 $$V_{t} = \phi^{ - 1} \left[ {H\left\{ {\frac{{\left( {n - 1} \right)\hat{\delta }_{(SELF)}^{2} }}{{\delta^{2} }}} \right\},\left( {n - 1} \right)} \right].$$

where $\hat{\theta }_{(SELF)} = \frac{{n\overline{x}\delta_{0}^{2} + \delta^{2} \theta_{0} }}{{\delta^{2} + n\delta_{0}^{2} }}$ and $\hat{\delta }_{(SELF)}^{2} = \frac{{\delta^{2} \delta_{0}^{2} }}{{\delta^{2} + n\delta_{0}^{2} }}$ are the Bayesian estimators using SELF for the population mean and variance, respectively, while using LLF, the Bayesian estimators for the population mean and variance are given as $\hat{\theta }_{{\left( {_{LLF} } \right)}} = \frac{{n\overline{z}\delta_{0}^{2} + \delta^{2} \theta_{0} }}{{\delta^{2} + n\delta_{0}^{2} }} - \frac{C\prime }{2}\delta_{n}^{2}$ and $\hat{\delta }_{{\left( {LLF} \right)}}^{2} = \frac{{\delta^{2} \delta_{0}^{2} }}{{\delta^{2} + n\delta_{0}^{2} }}$, the transform statistic under LLF for both the process mean and variance is mathematically discribed as:

14 $$U_{t} = \frac{{\hat{\theta }_{(LLF)} - \theta }}{{{\raise0.7ex\hbox{$\delta $} \!\mathord{\left/ {\vphantom {\delta {\sqrt n }}}\right.\kern-0pt} \!\lower0.7ex\hbox{${\sqrt n }$}}}}$$

and

15 $$V_{t} = \phi^{ - 1} \left[ {H\left\{ {\frac{{\left( {n - 1} \right)\hat{\delta }_{(LLF)}^{2} }}{{\delta^{2} }}} \right\},\left( {n - 1} \right)} \right]$$

where $H\left( {n,\nu } \right)$ is a chi-square distribution characterized by $\nu$ degrees of freedom, and $\phi^{ - 1}$ denotes the inverse of the standard normal distribution function. The computations for EWMA EWMA statistics regarding both the process mean and variance are outlined as follows:

16 $$P_{t(LF)} = \lambda U_{t(LF)} + \left( {1 - \lambda } \right)P_{t - 1(LF)}$$

17 $$Q_{t(LF)} = \lambda V_{t(LF)} + \left( {1 - \lambda } \right)V_{t - 1(LF)}$$

In this context, $P_{0}$ and $Q_{0}$ represent the initial values for the EWMA sequences *P*_*t*_ and *Q*_*t*_, respectively, with $\lambda$ (a constant within the range [0, 1]) denoting the smoothing constant. *P*_*t*_ and *Q*_*t*_ are also mutually independent because of the independence of *P*_*t*_ and *Q*_*t*_. When considering an in-control process, both *P*_*t*_ and *Q*_*t*_ follow normal distributions, each with a mean of zero and variances of $\delta_{{P_{t} }}^{2}$ and $\delta_{{Q_{t} }}^{2}$, respectively. This is defined as follows

18 $$\delta_{{P_{t} }}^{2} = \delta_{{Q_{t} }}^{2} = \frac{\lambda }{\lambda - 1}\left[ {1 - \left( {1 - \lambda } \right)^{2t} } \right],\quad for\,t - 1,2,..$$

The plotting statistics, Bayesian Max-EWMA for jointly monitoring using $P_{t(LF)}$ and $Q_{t(LF)}$ is mathematically defined

19 $$A_{t} = Max\left( {\left| {P_{t(LF)} } \right|,\left| {Q_{t(LF)} } \right|} \right),$$

For $$t = 1,2,..$$

As the Bayesian Max-EWMA statistic is a positive value, we required to plot only the upper control limit for jointly monitoring the process mean and variance. If the plotting statistic $A_{t}$ within the UCL, then the process is in control and if the $A_{t}$ cross the UCL, the process is out of control.

## Results and discussion

In this analysis, Tables 1, 2, 3 and 4 serve as a central platform for presenting the outcomes derived from the application of the Bayesian Max-EWMA CC for the Weibull process. This study undertakes a rigorous examination, specifically focusing on the influence of two distinct LFs designed to emphasize the significance of the posterior distribution. Importantly, these assessments are conducted within the framework of informative priors, introducing prior knowledge and beliefs into the analytical process. To ensure the reliability and robustness of our statistical conclusions, a substantial replication of 50, 000 replicates is employed for the calculation of both the ARL and SDRL. Furthermore, we exercise precision by carefully selecting smoothing constants, namely λ values of 0.10 and 0.25, which fine-tune our analysis and enable us to evaluate the performance of the Bayesian Max-EWMA CC method under diverse conditions. Furthermore, this study expands its scope and examines an extensive range of combinations with variance shift values (b) covering values from 0.25 to 3.00 and mean shift values (a) ranging from 0.00 to 3.00. This comprehensive analysis allows us to evaluate the performance of the Bayesian Max-EWMA CC approach, which specifically aims to comprehensively monitor process variance and mean simultaneously. The results of our study clearly show how sensitively the method can detect deviations from the standard within production processes. This demonstrates its enormous potential as a useful and trustworthy tool for continuous quality control and monitoring in a variety of industrial environments. It is important to ensure that the calculated plot statistic Si remains below the UCLi at all times. Each trial ends when the plot statistic exceeds the UCLi, indicating a significant change in the process mean and standard deviation. Changes in the parameters of the Weibull distribution are related to these variations. In particular, we analyze shifts in scale parameters ranging from 0.0 to 5.00 and shifts in shape parameters ranging from 0.25 to 4.00The process initially follows a normal distribution N(0, 1) and remains within the control limits when it comes to the Weibull distribution with parameters W(1, 1.5). The ARL under control conditions (ARL0) for the two different cases of the smoothing constant λ = 0.10 and 0.25 was found to be 370. The results shown in Tables 1 and 2 provide strong evidence for the effectiveness of the Bayesian Max-EWMA CC, particularly when used in conjunction with the SELF for the posterior distribution. Maintaining process stability and product quality depends on the combined approach's exceptional ability to simultaneously detect shifts in both process mean and variance. The results show a remarkable trend: the ARLs continuously decrease as the magnitude of the mean shift increases. Similarly, ARLs decrease when variance shifts occur. These recurring trends strongly suggest that the Bayesian Max-EWMA CC has the important ability to detect process changes in a timely manner and indicate what is needed for process control and early intervention. Due to these properties, it is an extremely valuable tool for thorough monitoring of production processes and ensures timely detection and correction of deviations from established standards. Ultimately, the use of these CCs improves process effectiveness and product quality, making them a valuable asset in a variety of industries. For example, if you look at the ARL results. The resulting ARL values for these shifts are as follows: 369.15, 80.19, 24.05, 13.71, 10.14, 8.16, 7.12, 4.88, and 4.13. Interestingly, the corresponding ARL values noticeably decrease with increasing displacement magnitude. This result shows the extent to which the proposed Bayesian Max-EWMA CC can be used to quickly identify changes in the shape parameter. The ability of the CC to quickly detect even small deviations from the process mean suggests that it is very sensitive. This means that these changes can be responded to quickly, which is critical to maintaining the consistency and quality of the process. The effects of changing the value of the shape parameter from a = 1.50 to 4.00 while maintaining the scaling parameter values ​are similar. The resulting ARL values are as follows: 369.15, 24.91, 16.92, 7.06, 5.80, 3.60, 2.60, 2.11, and 1.50. These ARL values show a clear trend: the ARL values sharply decline as the shape parameter deviates from the baseline value of 1. This pattern highlights how well the suggested Bayesian Max-EWMA CC performs in quickly identifying changes in process variance. Moreover, it is noteworthy that when examining the performance of the proposed Bayesian Max-EWMA CC in Table 2, we find that the CC becomes less effective as the smoothing constant increases. This observation suggests that in specific scenarios, opting for a lower smoothing constant might be more advantageous in achieving optimal performance. Similarly, Tables 3 and 4 present the ARL outcomes of the Bayesian Max-EWMA CC using the LLF with a consistent λ = 0.25 and *n* = 5. Across various trials involving shifts in the shape parameter ranging from 1.50 to 5.00 and corresponding shifts in the scale parameter fixed at 1.0, the resulting ARL values were 370.09, 20.34, 6.34, 2.81, 1.99, 1.57 and 1.17. These findings highlight a clear trend: as the magnitude of process shifts increases, the ARL values exhibit a rapid decrease, underscoring the exceptional accuracy of the proposed Max-EWMA CC in swiftly detecting shifts in both process mean and variance. Moreover, it is essential to note that the efficiency of the proposed CC for the simultaneous monitoring of the process mean and variance is influenced by the sample size. Across all the tables, a consistent pattern emerges: as the sample size increases, the corresponding ARL values decrease, indicating the enhanced effectiveness of the suggested CC in promptly identifying deviations from the expected process parameters. The following simulation steps have been considered for the calculations of ARLs and SDRLs.

**Table 1:** Run length profile of Bayesian Max-EWMA control chart as a result of a shift in Weibull parameters *W*(1, 1.5) with $\gamma$ = 0.10 and considering subgroup size as 3, 5, and 7.

$\alpha$	*n*	Shape parameter = $\lambda$											
		0.50	1.00	1.25	1.50	1.75	2.00	2.25	2.50	2.75	3.00	4.00	5.00
		*ARL* (*SDRL*)	*ARL* (*SDRL*)	*ARL* (*SDRL*)	*ARL* (*SDRL*)	*ARL* (*SDRL*)	*ARL* (*SDRL*)	*ARL* (*SDRL*)	*ARL* (*SDRL*)	*ARL* (*SDRL*)	*ARL* (*SDRL*)	*ARL* (*SDRL*)	*ARL* (*SDRL*)
0.25	(µ,σ)	(−1.51,2.04)	(−1.49,0.89)	(−1.45,0.72)	(−1.42,0.61)	(−1.40,0.53)	(−1.38,0.47)	(−1.36,0.42)	(−1.35,0.38)	(−1.33,0.35)	(−1.32,0.32)	(−1.29,0.25)	(−1.28,0.20)
	3	3.25 (1.23)	3.47 (0.76)	3.53 (0.65)	3.59 (0.60)	3.63 (0.56)	3.69 (0.53)	3.75 (0.51)	3.79 (0.46)	3.84 (0.43)	3.90 (0.36)	3.96 (0.30)	3.95 (0.22)
	5	2.52 (0.81)	2.69 (0.56)	2.77 (0.49)	2.84 (0.42)	2.90 (0.35)	2.93 (0.30)	2.97 (0.22)	2.98 (0.17)	3.00 (0.12)	3.00 (0.09)	2.99 (0.09)	2.96 (0.18)
	7	2.18 (0.62)	2.27 (0.45)	2.29 (0.45)	2.32 (0.47)	2.35 (0.47)	2.39 (0.48)	2.45 (0.49)	2.49 (0.49)	2.56 (0.49)	2.60 (0.48)	2.67 (0.46)	2.45 (0.49)
0.50	(µ,σ)	(−0.68,3.16)	(−0.87,1.11)	(−0.84,0.90)	(−0.81,0.76)	(−0.78,0.66)	(−0.76,0.58)	(−0.74,0.53)	(−0,72,0.48)	(−0.71,0.44)	(−0.70,0.40)	(−0.66,0.31)	(−0.64,0.25)
	3	2.67 (1.15)	6.38 (2.38)	6.61 (2.12)	6.94 (1.98)	7.22 (1.83)	7.33 (1.64)	7.47 (1.59)	7.49 (1.48)	7.33 (1.40)	7.05 (1.34)	5.97 (1.17)	5.16 (0.90)
	5	1.32 (0.46)	4.78 (1.53)	4.89 (1.31)	5.07 (1.19)	5.24 (1.09)	5.30 (0.98)	5.33 (0.91)	5.20 (0.86)	4.96 (0.80)	4.67 (0.78)	3.87 (0.60)	3.38 (0.51)
	7	1.63 (0.57)	3.95 (1.13)	4.06 (0.98)	4.20 (0.89)	4.33 (0.81)	4.35 (0.71)	4.35 (0.67)	4.21 (0.64)	4.00 (0.63)	3.71 (0.59)	3.09 (0.40)	2.83 (0.38)
0.75	(µ,σ)	(0.01,4.18)	(−0.42,1.30)	(−0.39,1.05)	(−0.36, 0.88)	(−0.34,0.77)	(−0.32, 0.68)	(−0.29, 0.61)	(−0.28,0.56)	(−0.26,0.51)	(−0.25,0.47)	(−0.21,0.37)	(−0.18,0.30)
	3	1.98 (0.88)	13.17 (7.45)	19.84 (12.19)	24.29 (14.79)	25.45 (14.48)	21.41 (10.90)	17.09 (7.96)	13.76 (5.55)	11.31 (4.07)	9.76 (3.08)	7.10	5.87 (1.18)
	5	1.49 (0.57)	9.38 (4.40)	13.36 (6.90)	15.53 (7.53)	14.69 (6.15)	11.76 (4.33)	9.18 (2.93)	7.63 (2.16)	6.46 (1.62)	5.73 (1.28)	4.44 (0.79)	3.79 (0.60)
		1.24 (0.43)	7.50 (3.16)	10.48 (4.85)	11.79 (5.05)	11.03 (3.91)	8.75 (2.63)	6.81 (1.85)	5.74 (1.37)	4.95 (1.02)	4.45 (0.86)	3.51 (0.57)	3.05 (0.37)
1.0	(µ,σ)	(0.61,5.00)	(−0.05,1.47)	(−0.03,1.18)	(0.00, 1.00)	(0.03,0.87)	(0.05,0.77)	(0.07,0.69)	(0.09,0.63)	(0.11,0.58)	(0.12,0.54)	(0.16,0.41)	(0.19,0.34)
	3	1.64 (0.72)	13.49 (8.07)	54.86 (45.68)	370.93 (366.30)	175.64 (164.11)	56.06 (43.89)	25.21 (14.91)	19.93 (10.69)	15.17 (6.67)	12.85 (5.03)	8.04 (2.16)	6.55 (1.44)
	5	1.26 (0.47)	8.48 (4.10)	31.98 (24.00)	369.15 (363.70)	80.19 (69.05)	24.05 (14.24)	13.71 (5.98)	10.14 (3.62)	8.16 (2.47)	7.12 (1.95)	4.88 (0.95)	4.13 (0.69)
	10	1.10 (0.31)	6.53 (2.80)	23.33 (15.76)	370.78 (366.11)	49.18 (38.50)	16.04 (7.92)	9.72 (3.51)	7.43 (2.19)	6.17 (1.55)	5.39 (1.23)	3.84 (0.65)	3.28 (0.50)
1.25	(µ,σ)	(1.17,5.69)	(0.28,1.65)	(0.29,1.31)	(0.32,1.11)	(0.35,0.96)	(0.37,0.86)	(0.39,0.77)	(0.41,0.70)	(0.43, 0.64)	(0.45,0.60)	(0.49,0.46)	(0.52,0.38)
	3	1.46 (0.63)	8.55 (4.46)	17.12 (10.76)	24.91 (17.03)	25.22 (16.24)	23.21 (13.62)	20.39 (10.21)	17.54 (7.84)	14.97 (5.75)	13.28 (4.62)	9.10 (2.49)	7.32 (1.74)
	5	1.17 (0.38)	5.69 (2.38)	11.35 (5.94)	16.92 (9.87)	16.24 (8.73)	14.70 (6.86)	12.65 (4.90)	10.64 (3.45)	9.08 (2.58)	8.11 (2.14)	6.03 (1.38)	6.63 (1.65)
	10	1.05 (0.23)	4.49 (1.66)	8.81 (4.02)	13.14 (6.87)	12.38 (5.88)	11.23 (4.47)	9.62 (3.14)	8.18 (2.26)	6.98 (1.70)	6.26 (1.43)	4.32 (0.81)	3.59 (0.59)

v$\alpha$	*N*	Shape parameter = $\lambda$											
		0.50	1.00	1.25	1.50	1.75	2.00	2.25	2.50	2.75	3.00	4.00	5.00
		*ARL* (*SDRL*)	*ARL* (*SDRL*)	*ARL* (*SDRL*)	*ARL* (*SDRL*)	*ARL* (*SDRL*)	*ARL* (*SDRL*)	*ARL* (*SDRL*)	*ARL* (*SDRL*)	*ARL* (*SDRL*)	*ARL* (*SDRL*)	*ARL* (*SDRL*)	*ARL* (*SDRL*)
1.50	(µ, σ)	(1.67, 6.26)	(0.58, 1.86)	(0.58, 1.44)	(0.61, 1.21)	(0.64, 1.05)	(0.66, 0.94)	(0.68, 0.84)	(0.70, 0.77)	(0.72, 0.71)	(0.74, 0.65)	(0.79, 0.51)	(0.82, 0.41)
	3	1.35 (0.56)	5.69 (2.63)	8.64 (4.27)	9.82 (4.76)	9.54 (4.21)	9.15 (3.65)	8.82 (3.07)	8.41 ( 2.68)	8.13 (2.39)	7.73 (1.99)	6.89 (1.33)	6.35 (1.00)
	5	1.10 ( 0.31)	4.00 ( 1.49)	6.23 ( 2.54)	7.06 ( 2.89)	6.80 ( 2.50)	6.55 ( 2.13)	6.29 ( 1.81)	6.04 ( 1.57)	5.76 ( 1.35)	5.57 ( 1.17)	4.97 ( 0.78)	12.32 ( 5.02)
	7	1.02 ( 0.16)	3.31 ( 1.12)	5.09 ( 1.86)	5.80 ( 2.16)	5.58 ( 1.86)	3.02 ( 0.99)	5.11 ( 1.31)	4.96 ( 1.16)	4.77 ( 0.99)	4.40 ( 0.69)	4.08 ( 0.59)	3.67 ( 0.52)
2.00	(µ, σ)	(2.57, 7.16)	(1.16, 2.43)	(1.11, 1.70)	(1.13, 1.41)	(1.15, 1.23)	(1.18, 1.09)	(1.20, 0.98)	(1.23, 0.89)	(1.25, 0.82)	(1.26, 0.76)	(1.32, 0.59)	(1.36, 0.48)
	3	1.24 (0.46)	3.53 (1.47)	4.47 (1.81)	4.70 (1.76)	4.64 (1.58)	4.49 (1.37)	4.40 (1.21)	4.26 (1.04)	4.16 (0.94)	4.13 (0.86)	3.87 (0.64)	3.75 (0.54)
	5	1.05 ( 0.23)	2.44 ( 0.82)	3.41 ( 1.15)	3.60 ( 1.17)	3.52 ( 1.01)	2.26 ( 0.47)	3.34 ( 0.78)	3.26 ( 0.69)	3.19 ( 0.62)	3.16 ( 0.56)	3.02 ( 0.40)	2.96 ( 0.29)
	7	1.01 ( 0.11)	2.07 ( 0.63)	2.89 ( 0.89)	3.02 ( 0.88)	2.97 ( 0.79)	2.89 ( 0.69)	2.83 ( 0.62)	2.77 ( 0.57)	2.71 ( 0.54)	2.68 ( 0.52)	2.57 ( 0.49)	2.46 ( 0.49)
2.50	(µ, σ)	(3.33, 7.82)	(1.74, 3.18)	(1.59, 2.05)	(1.59, 1.61)	(1.62, 1.39)	(1.64, 1.23)	(1.67, 1.11)	(1.69, 1.01)	(1.72, 0.93)	(1.73, 0.86)	(1.80, 0.67)	(1.84, 0.55)
	3	1.18 (0.41)	2.26 (0.94)	3.14 (1.18)	3.31 (1.13)	3.25 (1.00)	3.18 (0.89)	3.11 (0.77)	3.06 (0.71)	3.01 (0.64)	2.99 (0.60)	2.88 (0.46)	2.82 (0.46)
	5	1.03 ( 0.18)	1.74 ( 0.63)	2.45 ( 0.78)	2.60 ( 0.77)	2.55 ( 0.68)	2.49 ( 0.60)	2.43 ( 0.55)	2.40 ( 0.52)	2.33 ( 0.49)	2.31 ( 0.46)	2.16 ( 0.37)	2.08 ( 0.27)
	7	1.00 ( 0.07)	1.49 ( 0.54)	2.10 ( 0.62)	2.25 ( 0.59)	2.22 ( 0.52)	2.16 ( 0.44)	2.13 ( 0.38)	2.09 ( 0.32)	2.05 ( 0.27)	2.04 ( 0.23)	2.00 ( 0.08)	2.00 ( 0.02)
3.00	(µ, σ)	(4.00, 8.33)	(2.35, 4.00)	(2.08, 2.58)	(2.03, 1.86)	(2.04, 1.55)	(2.07, 1.37)	(2.10, 1.24)	(2.12, 1.13)	(2.15, 1.04)	(2.17, 0.96)	(2.24, 0.75)	(2.29, 0.61)
	3	1.13 (0.35)	1.75 (0.75)	2.38 (0.92)	2.64 (0.88)	2.63 (0.76)	2.58 (0.69)	2.51 (0.61)	2.49 (0.61)	2.43 (0.54)	2.40 (0.51)	2.28 (0.45)	2.16 (0.36)
	5	1.02 (0.14)	1.37 (0.51)	1.88 (0.63)	2.11 (0.62)	2.11 (0.52)	2.08 (0.43)	2.05 (0.37)	2.03 (0.32)	2.01 (0.28)	2.00 (0.23)	1.99 (0.10)	1.99 (0.05)
	7	1.00 (0.06)	1.18 (0.39)	1.75 (0.54)	1.84 (0.52)	1.86 (0.46)	1.85 (0.42)	1.85 (0.38)	1.85 (0.36)	1.85 (0.35)	1.85 (0.35)	1.87 (0.33)	1.87 (0.32)
4.00	(µ, σ)	(5.12, 9.07)	(3.61, 5.53)	(3.15, 3.99)	(2.93, 2.81)	(2.86, 2.07)	(2.86, 1.68)	(2.88, 1.48)	(2.91, 1.35)	(2.94, 1.24)	(2.96, 1.15)	(3.04, 0.89)	(3.10, 0.73)
	3	1.10 (0.31)	1.33 (0.54)	1.61 (0.67)	1.86 (0.71)	1.97 (0.62)	1.94 (0.71)	1.94 (0.70)	1.95 (0.40)	1.95 (0.35)	1.94 (0.32)	1.96 (0.20)	1.97 (0.17)
	5	1.01 (0.11)	1.10 (0.30)	1.26 (0.45)	1.50 (0.55)	1.59 (0.53)	1.59 (0.50)	1.59 (0.49)	1.58 (0.49)	1.56 (0.49)	1.55 (0.49)	1.48 (0.49)	1.42 (0.49)
	7	1.00 (0.03)	1.02 (0.15)	1.05 (0.22)	1.29 (0.46)	1.34 (0.48)	1.32 (0.46)	1.29 (0.45)	1.24 (0.43)	1.20 (0.40)	1.17 (0.37)	1.07 (0.26)	1.02 (0.15)

**Table 2:** Run length profile of Bayesian Max-EWMA control chart as a result of a shift in Weibull parameters *W*(1, 1.5) with $\gamma$=0.25 and considering subgroup size as 3, 5, and 7.

$\alpha$	*n*	Shape parameter = $\lambda$											
		0.50	1.00	1.25	1.50	1.75	2.00	2.25	2.50	2.75	3.00	4.00	5.00
		*ARL* (*SDRL*)	*ARL* (*SDRL*)	*ARL* (*SDRL*)	*ARL* (*SDRL*)	*ARL* (*SDRL*)	*ARL* (*SDRL*)	*ARL* (*SDRL*)	*ARL* (*SDRL*)	*ARL* (*SDRL*)	*ARL* (*SDRL*)	*ARL* (*SDRL*)	*ARL* (*SDRL*)
0.25	(µ, σ)	(−1.51, 2.04)	(−1.49, 0.89)	(−1.45, 0.72)	(−1.42, 0.61)	(−1.40, 0.53)	(−1.38, 0.47)	(−1.36, 0.42)	(−1.35, 0.38)	(−1.33, 0.35)	(−1.32, 0.32)	(−1.29, 0.25)	(−1.28, 0.20)
	3	2.46 (1.17)	2.74 (0.79)	2.79 (0.71)	2.84 (0.65)	2.87 (0.58)	2.93 (0.54)	2.98 (0.53)	2.99 (0.43)	3.03 (0.39)	3.05 (0.37)	3.05 (0.31)	3.01 (0.27)
	5	1.87 (0.77)	2.10 (0.46)	2.12 (0.39)	2.12 (0.34)	2.12 (0.33)	2.11 (0.32)	2.12 (0.33)	2.11 (0.32)	2.12 (0.33)	2.12 (0.32)	2.09 (0.29)	2.03 (0.19)
	7	1.60 (0.62)	1.84 (0.42)	1.90 (0.33)	1.95 (0.25)	1.97 (0.17)	1.99 (0.10)	1.99 (0.07)	1.99 (0.04)	1.99 (0.03)	1.99 (0.02)	1.99 (0.05)	1.98 (0.10)
0.50	(µ, σ)	(−0.68, 3.16)	(−0.87, 1.11)	(−0.84, 0.90)	(−0.81, 0.76)	(−0.78, 0.66)	(−0.76, 0.58)	(−0.74, 0.53)	(−0, 72, 0.48)	(−0.71, 0.44)	(−0.70, 0.40)	(−0.66, 0.31)	(−0.64, 0.25)
	3	1.96 (1.00)	5.74 (2.99)	6.22 (2.92)	6.78 (3.04)	7.28 (3.18)	7.58 (3.02)	7.89 (3.09)	7.92 (3.01)	7.67 (2.80)	7.19 (2.54)	5.61 (1.82)	4.52 (1.22)
	5	1.48 (0.63)	3.97 (1.66)	4.15 (1.55)	4.38 (1.69)	4.52 (1.39)	4.57 (1.28)	4.54 (1.19)	4.44 (1.10)	4.18 (1.00)	3.85 (0.91)	3.09 (0.65)	2.65 (0.53)
	7	1.25 (0.46)	3.18 (1.19)	3.29 (1.07)	3.42 (0.99)	3.54 (0.93)	3.52 (0.82)	3.49 (0.75)	3.37 (0.71)	3.16 (0.66)	2.93 (0.60)	2.36 (0.49)	2.06 (0.26)
0.75	(µ, σ)	(0.01, 4.18)	(−0.42, 1.30)	(−0.39, 1.05)	(−0.36, 0.88)	(−0.34, 0.77)	(−0.32, 0.68)	(−0.29, 0.61)	(−0.28, 0.56)	(−0.26, 0.51)	(−0.25, 0.47)	(−0.21, 0.37)	(−0.18, 0.30)
	3	1.51 (0.71)	13.13 (9.90)	26.32 (22.10)	45.30 (39.60)	59.18 (53.36)	50.09 (43.58)	32.10 (26.23)	22.55 (17.06)	15.92 (10.83)	12.31 (7.34)	7.35 (3.11)	5.45 (1.79)
	5	1.17 (0.40)	8.49 (5.55)	15.71 (11.85)	22.41 (17.22)	23.19 (17.42)	16.22 (10.93)	10.61 (5.99)	7.91 (3.78)	6.18 (2.50)	5.22 (1.83)	3.67 (0.91)	3.01 (0.62)
		1.07 (0.26)	6.49 (3.69)	11.26 (7.79)	14.61 (9.95)	13.90 (8.94)	9.54 (5.13)	6.59 (2.87)	4.23 (1.32)	4.25 (1.31)	3.69 (0.99)	2.75 (0.60)	2.29 (0.46)
1.0	(µ, σ)	(0.61, 5.00)	(−0.05, 1.47)	(−0.03, 1.18)	(0.00, 1.00)	(0.03, 0.87)	(0.05, 0.77)	(0.07, 0.69)	(0.09, 0.63)	(0.11, 0.58)	(0.12, 0.54)	(0.16, 0.41)	(0.19, 0.34)
	3	1.31 (0.55)	12.51 (9.61)	61.46 (57.13)	370.87 (367.43)	348.36 (344.16)	142.09 (138.02)	73.03 (66.94)	39.54 (33.40)	25.911 (20.49)	19.30 (13.62)	8.89 (4.45)	6.42 (2.41)
	5	1.09 (0.29)	7.58 (4.94)	39.57 (35.42)	370.89 (365.34)	173.67 (170.29)	47.57 (42.11)	20.20 (14.96)	12.35 (7.48)	8.89 (4.60)	7.14 (3.19)	4.17 (1.20)	3.36 (0.77)
	10	1.02 (0.15)	5.56 (3.14)	28.26 (24.46)	370.87 (364.22)	105.35 (100.46)	25.09 (19.74)	11.31 (6.90)	7.48 (3.61)	5.68 (2.26)	1.94 (0.22)	3.06 (0.71)	2.57 (0.55)
1.25	(µ, σ)	(1.17, 5.69)	(0.28, 1.65)	(0.29, 1.31)	(0.32, 1.11)	(0.35, 0.96)	(0.37, 0.86)	(0.39, 0.77)	(0.41, 0.70)	(0.43, 0.64)	(0.45, 0.60)	(0.49, 0.46)	(0.52, 0.38)
	3	1.21 (0.45)	7.12 (4.76)	17.29 (13.83)	31.77 (27.64)	40.80 (35.39)	43.02 (37.31)	22.66 (16.88)	36.07 (30.02)	28.31 (22.21)	23.14 (16.89)	11.16 (6.11)	7.56 (3.23)
	5	1.05 (0.21)	4.61 (2.54)	10.81 (7.48)	20.05 (16.16)	21.90 (17.12)	21.10 (15.69)	17.50 (12.17)	13.62 (8.24)	10.41 (5.50)	8.67 (4.10)	4.97 (1.61)	3.09 (0.65)
	10	1.01 (0.10)	3.56 (1.70)	8.01 (5.09)	14.35 (10.79)	15.09 (10.87)	13.52 (8.89)	10.94 (6.17)	8.55 (4.07)	6.74 (2.70)	5.71 (2.06)	3.56 (0.92)	2.82 (0.62)

$\alpha$	*N*	Shape parameter = $\lambda$											
		0.50	1.00	1.25	1.50	1.75	2.00	2.25	2.50	2.75	3.00	4.00	5.00
		*ARL* (*SDRL*)	*ARL* (*SDRL*)	*ARL* (*SDRL*)	*ARL* (*SDRL*)	*ARL* (*SDRL*)	*ARL* (*SDRL*)	*ARL* (*SDRL*)	*ARL* (*SDRL*)	*ARL* (*SDRL*)	*ARL* (*SDRL*)	*ARL* (*SDRL*)	*ARL* (*SDRL*)
1.50	(µ, σ)	(1.67, 6.26)	(0.58, 1.86)	(0.58, 1.44)	(0.61, 1.21)	(0.64, 1.05)	(0.66, 0.94)	(0.68, 0.84)	(0.70, 0.77)	(0.72, 0.71)	(0.74, 0.65)	(0.79, 0.51)	(0.82, 0.41)
	3	1.16 (0.39)	4.40 (2.52)	7.48 (4.96)	9.34 (6.31)	9.79 (6.21)	9.76 (5.90)	9.46 (5.33)	9.04 (4.81)	8.52 (4.23)	8.07 (3.67)	6.87 (2.38)	5.98 (1.69)
	5	1.03 ( 0.18)	3.10 (1.47)	5.16 (2.85)	6.29 (3.53)	6.20 (3.27)	6.10 (2.96)	5.75 (2.48)	5.46 (2.16)	5.22 (1.86)	4.88 (1.57)	3.62 (0.78)	3.64 (0.73)
	7	1.00 (0.07)	2.51 (1.05)	4.10 (1.96)	4.97 (2.47)	4.82 (2.17)	4.60 (1.92)	4.41 (1.62)	4.17 (1.35)	3.95 (1.18)	3.75 (1.00)	3.24 (0.64)	2.85 (0.51)
2.00	(µ, σ)	(2.57, 7.16)	(1.16, 2.43)	(1.11, 1.70)	(1.13, 1.41)	(1.15, 1.23)	(1.18, 1.09)	(1.20, 0.98)	(1.23, 0.89)	(1.25, 0.82)	(1.26, 0.76)	(1.32, 0.59)	(1.36, 0.48)
	3	1.10 (0.32)	2.42 (1.22)	3.49 (1.76)	3.82 (1.88)	2.99 (1.14)	3.70 (1.51)	3.60 (1.32)	3.47 (1.16)	3.40 (1.06)	3.33 (0.97)	3.11 (0.70)	2.98 (0.54)
	5	1.01 (0.13)	1.80 (0.77)	2.59 (1.09)	2.84 (1.16)	2.81 (1.03)	2.71 (0.90)	2.64 (0.80)	2.55 (0.70)	2.49 (0.63)	2.58 (0.64)	2.27 (0.46)	2.15 (0.36)
	7	1.00 (0.04)	1.52 (0.60)	2.19 (0.83)	2.36 (0.84)	2.33 (0.76)	2.27 (0.66)	2.21 (0.58)	2.14 (0.49)	2.11 (0.44)	2.09 (0.39)	2.01 (0.21)	1.99 (0.11)
2.50	(µ, σ)	(3.33, 7.82)	(1.74, 3.18)	(1.59, 2.05)	(1.59, 1.61)	(1.62, 1.39)	(1.64, 1.23)	(1.67, 1.11)	(1.69, 1.01)	(1.72, 0.93)	(1.73, 0.86)	(1.80, 0.67)	(1.84, 0.55)
	3	1.07 (0.26)	1.70 ( 0.81)	2.34 (1.09)	2.59 (1.10)	2.56 (0.99)	2.51 (0.87)	2.46 (0.77)	2.41 (0.69)	2.34 (0.61)	2.32 (0.56)	2.18 (0.41)	2.10 ( 0.31)
	5	1.00 (0.08)	1.30 (0.50)	1.82 (0.73)	1.97 (0.73)	1.96 (0.60)	1.95 (0.59)	1.91 (0.53)	1.89 (0.48)	1.87 (0.43)	1.86 (0.41)	1.85 (0.35)	1.86 (0.34)
	7	1.00 (0.02)	1.14 (0.36)	1.54 (0.59)	1.68 (0.61)	1.90 (0.69)	1.65 (0.53)	1.62 ( 0.51)	1.60 (0.50)	1.57 (0.49)	1.57 (0.49)	1.47 (0.49)	1.40 (0.49)
3.00	(µ, σ)	(4.00, 8.33)	(2.35, 4.00)	(2.08, 2.58)	(2.03, 1.86)	(2.04, 1.55)	(2.07, 1.37)	(2.10, 1.24)	(2.12, 1.13)	(2.15, 1.04)	(2.17, 0.96)	(2.24, 0.75)	(2.29, 0.61)
	3	1.05 (0.23)	1.36 (0.60)	1.77 (0.81)	2.01 (0.83)	2.02 (0.75)	2.00 (0.68)	1.95 (0.60)	1.93 (0.54)	1.90 (0.50)	1.90 (0.46)	1.88 (0.36)	1.89 (0.31)
	5	1.00 (0.07)	1.18 (0.40)	1.39 (0.55)	1.57 (0.61)	1.57 (0.57)	1.55 (0.54)	1.52 (0.53)	1.51 (0.51)	1.47 (0.50)	1.45 (0.49)	1.35 (0.47)	1.25 (0.43)
	7	1.00 (0.01)	1.04 (0.19)	1.21 (0.42)	1.34 (0.49)	1.33 (0.48)	1.29 ( 0.45)	1.24 (0.43)	1.22 (0.41)	1.17 (0.38)	1.14 (0.35)	1.04 (0.21)	1.01 (0.12)
4.00	(µ, σ)	(5.12, 9.07)	(3.61, 5.53)	(3.15, 3.99)	(2.93, 2.81)	(2.86, 2.07)	(2.86, 1.68)	(2.88, 1.48)	(2.91, 1.35)	(2.94, 1.24)	(2.96, 1.15)	(3.04, 0.89)	(3.10, 0.73)
	3	1.03 (0.19)	1.16 (0.39)	1.26 (0.49)	1.42 (0.59)	1.48 (0.59)	1.47 (0.55)	1.43 (0.51)	1.41 (0.50)	1.38 (0.49)	1.36 (0.48)	1.27 (0.44)	1.19 (0.39)
	5	1.00 (0.04)	1.02 (0.16)	1.08 (0.27)	1.16 (0.38)	1.19 (0.40)	1.15 ( 0.36)	1.12 (0.33)	1.09 (0.29)	1.06 (0.25)	1.05 (0.21)	1.00 (0.09)	1.00 (0.03)
	7	1.00 (0.01)	1.00 (0.06)	1.02 (0.14)	1.05 (0.23)	1.06 (0.25)	1.04 (0.20)	1.02 (0.16)	1.01 (0.11)	1.00 (0.08)	1.00 (0.05)	1.27 (0.47)	1.00 (0.00)

**Table 3:** The ARL results of Bayesian Max-EWMA control chart as a result of a shift in Weibull parameters *W*(1, 1.5) with $\gamma$ = 0.10 and considering subgroup size as 3, 5, and 7 under LLF.

$\alpha$	*N*	Shape parameter = $\lambda$											
		0.50	1.00	1.25	1.50	1.75	2.00	2.25	2.50	2.75	3.00	4.00	5.00
		*ARL* (*SDRL*)	*ARL* (*SDRL*)	*ARL* (*SDRL*)	*ARL* (*SDRL*)	*ARL* (*SDRL*)	*ARL* (*SDRL*)	*ARL* (*SDRL*)	*ARL* (*SDRL*)	*ARL* (*SDRL*)	*ARL* (*SDRL*)	*ARL* (*SDRL*)	*ARL* (*SDRL*)
0.25	(µ, σ)	(−1.51, 2.04)	(−1.49, 0.89)	(−1.45, 0.72)	(−1.42, 0.61)	(−1.40, 0.53)	(−1.38, 0.47)	(−1.36, 0.42)	(−1.35, 0.38)	(−1.33, 0.35)	(−1.32, 0.32)	(−1.29, 0.25)	(−1.28, 0.20)
	3	3.24 (1.24)	3.46 (0.75)	3.53 (0.66)	3.59 (0.60)	3.62 (0.56)	3.68 (0.53)	3.76 (0.49)	3.79 (0.47)	3.85 (0.42)	3.89 (0.37)	3.96 (0.24)	3.95 (0.23)
	5	2.53 (0.80)	2.70 (0.56)	2.77 (0.50)	2.84 (0.43)	2.90 (0.36)	2.94 (0.29)	2.97 (0.22)	2.98 (0.17)	2.99 (0.13)	3.00 (0.10)	2.99 (0.09)	2.96 (0.17)
	7	2.17 (0.64)	2.27 (0.45)	2.29 (0.45)	2.33 (0.47)	2.35 (0.47)	2.39 (0.48)	2.46 (0.49)	2.49 (0.49)	2.57 (0.49)	2.61 (0.48)	2.68 (0.46)	2.44 (0.49)
0.50	(µ, σ)	(−0.68, 3.16)	(−0.87, 1.11)	(−0.84, 0.90)	(−0.81, 0.76)	(−0.78, 0.66)	(−0.76, 0.58)	(−0.74, 0.53)	(−0.72, 0.48)	(−0.71, 0.44)	(−0.70, 0.40)	(−0.66, 0.31)	(−0.64, 0.25)
	3	2.65 (1.14)	6.41 (2.40)	6.70 (2.17)	6.91 (1.96)	7.21 (1.82)	7.41 (1.67)	7.50 (1.57)	7.53 (1.50)	7.32 (1.40)	7.03 (1.36)	8.38 (1.89)	5.19 (0.91)
	5	1.94 (0.71)	4.75 (1.50)	4.92 (1.31)	5.07 (1.22)	5.24 (1.09)	5.47 (1.47)	5.30 (0.90)	5.20 (0.86)	4.96 (0.82)	4.68 (0.78)	3.87 (0.61)	3.10 (0.36)
	7	1.64 (0.57)	3.94 (1.11)	4.06 (0.98)	4.18 (0.88)	4.34 (0.83)	4.35 (0.72)	4.35 (0.67)	4.21 (0.63)	3.99 (0.62)	3.72 (0.59)	3.09 (0.39)	2.83 (0.37)
0.75	(µ, σ)	(0.01, 4.18)	(−0.42, 1.30)	(−0.39, 1.05)	(−0.36, 0.88)	(−0.34, 0.77)	(−0.32, 0.68)	(−0.29, 0.61)	(−0.28, 0.56)	(−0.26, 0.51)	(−0.25, 0.47)	(−0.21, 0.37)	(−0.18, 0.30)
	3	1.99 (0.87)	13.27 (7.54)	19.93 (12.35)	24.68 (15.17)	25.30 (14.43)	21.44 (10.98)	16.99 (7.88)	13.86 (5.75)	11.28 (4.02)	9.75 (3.09)	7.15 (2.87)	5.87 (1.16)
	5	1.47 (0.57)	9.32 (4.39)	13.42 (6.97)	15.43 (7.74)	14.67 (6.20)	11.85 (4.34)	9.16 (2.94)	7.61 (7.67)	6.46 (1.61)	5.73 (1.28)	4.42 (0.78)	3.79 (0.59)
	7	1.25 (0.44)	7.47 (3.07)	10.52 (4.92)	11.77 (4.98)	9.65 (3.16)	8.69 (2.66)	6.80 (1.85)	5.72 (1.36)	4.95 (1.04)	4.43 (0.86)	3.50 (0.57)	3.04 (0.37)
1.0	(µ, σ)	(0.61, 5.00)	(−0.05, 1.47)	(−0.03, 1.18)	(0.00, 1.00)	(0.03, 0.87)	(0.05, 0.77)	(0.07, 0.69)	(0.09, 0.63)	(0.11, 0.58)	(0.12, 0.54)	(0.16, 0.41)	(0.19, 0.34)
	3	1.66 (0.73)	13.61 (8.24)	54.88 (46.29)	369.33 (367.12)	173.35 (160.14)	57.52 (45.17)	29.07 (18.42)	19.61 (10.19)	15.23 (6.89)	12.76 (5.05)	8.05 (2.16)	6.53 (1.46)
	5	1.27 (0.46)	8.47 (4.08)	31.78 (23.77)	370.89 (366.45)	79.18 (67.14)	24.21 (14.68)	13.64 (6.01)	10.17 (3.64)	8.21 (2.46)	7.13 (1.93)	4.89 (0.94)	4.14 (0.68)
	7	1.11 (0.32)	6.52 (2.76)	23.51 (15.64)	369.87 (360.22)	49.61 (38.56)	15.99 (7.87)	9.67 (3.48)	7.42 (2.17)	6.17 (1.59)	5.39 (1.23)	3.83 (0.65)	3.28 (0.49)
1.25	(µ, σ)	(1.17, 5.69)	(0.28, 1.65)	(0.29, 1.31)	(0.32, 1.11)	(0.35, 0.96)	(0.37, 0.86)	(0.39, 0.77)	(0.41, 0.70)	(0.43, 0.64)	(0.45, 0.60)	(0.49, 0.46)	(0.52, 0.38)
	3	1.47 (0.64)	8.56 (4.48)	17.12 (10.83)	25.07 (16.79)	25.43 (16.08)	23.33 (13.86)	20.32 (10.33)	17.52 (7.75)	15.06 (5.84)	13.33 (4.67)	10.08 (3.00)	7.28 (1.75)
	5	1.17 (0.38)	5.67 (2.38)	11.37 (5.86)	16.89 (10.07)	16.33 (8.78)	14.76 (6.96)	12.62 (4.85)	10.71 (3.48)	9.05 (2.55)	8.12 (2.13)	5.55 (1.17)	4.55 (0.82)
	7	1.05 (0.22)	4.50 (1.68)	8.83 (4.01)	13.31 (7.06)	12.58 (5.94)	11.15 (4.44)	9.70 (3.18)	8.19 (2.26)	6.99 (1.74)	6.22 (1.43)	4.33 (0.82)	3.60 (0.59)

$\alpha$	*n*	Shape parameter = $\lambda$											
		0.50	1.00	1.25	1.50	1.75	2.00	2.25	2.50	2.75	3.00	4.00	5.00
		*ARL* (*SDRL*)	*ARL* (*SDRL*)	*ARL* (*SDRL*)	*ARL* (*SDRL*)	*ARL* (*SDRL*)	*ARL* (*SDRL*)	*ARL* (*SDRL*)	*ARL* (*SDRL*)	*ARL* (*SDRL*)	*ARL* (*SDRL*)	*ARL* (*SDRL*)	*ARL* (*SDRL*)
1.50	(µ, σ)	(1.67, 6.26)	(0.58, 1.86)	(0.58, 1.44)	(0.61, 1.21)	(0.64, 1.05)	(0.66, 0.94)	(0.68, 0.84)	(0.70, 0.77)	(0.72, 0.71)	(0.74, 0.65)	(0.79, 0.51)	(0.82, 0.41)
	3	1.36 (0.56)	5.68 (2.59)	8.58 (4.26)	9.80 (4.77)	9.54 (9.54)	9.19 (3.63)	8.77 (3.12)	8.38 (2.65)	8.09 (2.35)	7.73 (2.00)	6.91 (1.33)	6.36 (1.01)
	5	1.11 (0.31)	3.98 (1.47)	6.23 (2.54)	7.05 (2.87)	6.80 (2.50)	6.56 (2.16)	6.29 (1.88)	6.02 (1.58)	5.81 (1.37)	5.54 (1.17)	5.20 (1.16)	4.47 (0.64)
	7	1.02 (0.16)	3.30 (1.10)	5.08 (1.84)	5.78 (2.14)	5.57 (1.86)	5.34 (1.56)	5.13 (1.33)	4.93 (1.15)	4.75 (1.00)	4.56 (0.86)	4.07 (0.59)	3.66 (0.52)
2.00	(µ, σ)	(2.57, 7.16)	(1.16, 2.43)	(1.11, 1.70)	(1.13, 1.41)	(1.15, 1.23)	(1.18, 1.09)	(1.20, 0.98)	(1.23, 0.89)	(1.25, 0.82)	(1.26, 0.76)	(1.32, 0.59)	(1.36, 0.48)
	3	1.24 (0.46)	2.20 (0.44)	4.51 (1.83)	4.68 (1.78)	4.63 (1.57)	4.49 (1.35)	4.38 (1.19)	4.25 (1.04)	4.14 (0.92)	4.12 (0.87)	3.88 (0.65)	3.74 (0.54)
	5	1.05 (0.23)	2.41 (0.82)	3.40 (1.13)	3.61 (1.16)	3.55 (1.02)	3.43 (0.89)	3.35 (0.78)	3.25 (0.69)	3.19 (0.62)	3.17 (0.57)	3.02 (0.40)	2.96 (0.29)
	7	1.01 (0.10)	1.62 (0.48)	2.10 (0.35)	2.44 (0.56)	2.79 (0.69)	3.15 (0.83)	3.51 ( 1.00)	3.50 (1.01)	4.20 (1.36)	4.53 (1.51)	5.64 (2.13)	2.46 (0.49)
2.50	(µ, σ)	(3.33, 7.82)	(1.74, 3.18)	(1.59, 2.05)	(1.59, 1.61)	(1.62, 1.39)	(1.64, 1.23)	(1.67, 1.11)	(1.69, 1.01)	(1.72, 0.93)	(1.73, 0.86)	(1.80, 0.67)	(1.84, 0.55)
	3	1.18 (0.41)	2.29 (0.95)	3.14 (1.17)	3.31 ( 1.13)	3.27 (1.01)	3.20 (0.88)	3.12 (0.79)	3.08 (0.71)	3.02 (0.64)	2.98 (0.60)	2.87 (0.51)	2.84 ( 0.39)
	5	1.05 (0.22)	1.75 (0.64)	2.45 ( 0.78)	2.58 ( 0.75)	2.55 (0.67)	2.50 (0.60)	2.43 (0.55)	2.39 (0.52)	2.34 (0.48)	2.31 (0.47)	2.17 (0.38)	2.08 (0.27)
	7	1.00 (0.07)	1.17 (0.38)	2.09 (0.60)	2.24 (0.58)	2.65 (0.77)	2.72 (0.80)	2.91 (0.89)	3.08 (0.96)	3.19 ( 1.02)	2.04 (0.23)	2.00 (0.08)	2.00 (0.01)
3.00	(µ, σ)	(4.00, 8.33)	(2.35, 4.00)	(2.08, 2.58)	(2.03, 1.86)	(2.04, 1.55)	(2.07, 1.37)	(2.10, 1.24)	(2.12, 1.13)	(2.15, 1.04)	(2.17, 0.96)	(2.24, 0.75)	(2.29, 0.61)
	3	1.14 (0.37)	1.76 (0.75)	2.40 (0.93)	2.63 (0.88)	2.63 (0.78)	2.58 (0.68)	2.51 (0.62)	2.48 (0.57)	2.43 (0.53)	2.39 (0.51)	2.27 (0.45)	2.17 (0.38)
	5	1.02 ( 0.15)	1.36 (0.51)	1.88 (0.63)	2.22 (0.70)	2.48 (0.79)	2.33 (0.86)	2.06 (0.47)	2.03 (0.32)	2.01 (0.27)	2.01 (0.22)	1.99 (0.12)	1.99 (0.06)
	7	1.00 (0.05)	1.17 (0.38)	1.63 (0.54)	1.83 (0.52)	1.86 (0.46)	1.85 (0.41)	1.84 (0.38)	1.85 (0.36)	1.85 (0.35)	1.85 (0.35)	1.86 (0.33)	1.99 ( 0.01)
4.00	(µ, σ)	(5.12, 9.07)	(3.61, 5.53)	(3.15, 3.99)	(2.93, 2.81)	(2.86, 2.07)	(2.86, 1.68)	(2.88, 1.48)	(2.91, 1.35)	(2.94, 1.24)	(2.96, 1.15)	(3.04, 0.89)	(3.10, 0.73)
	3	1.10 (0.31)	1.33 (0.53)	1.59 (0.66)	1.88 (0.71)	1.98 (0.62)	1.98 (0.52)	1.97 (0.45)	1.96 ( 0.39)	1.95 ( 0.35)	1.94 (0.31)	1.96 (0.21)	1.97 (0.16)
	5	1.01 (0.12)	1.09 (0.30)	1.27 (0.46)	1.48 (0.54)	1.59 (0.53)	1.59 (0.50)	1.58 (0.49)	1.58 (0.49)	1.55 (0.51)	1.53 (0.49)	1.48 (0.49)	1.42 (0.49)
	7	1.00 (0.03)	1.02 (0.16)	1.12 (0.32)	1.28 ( 0.45)	1.34 (0.47)	1.32 (0.46)	1.27 (0.44)	1.23 (0.42)	1.21 (0.40)	1.17 (0.38)	1.07 (0.26)	1.02 (0.16)

**Table 4:** The ARL results of Bayesian Max-EWMA control chart as a result of a shift in Weibull parameters *W(*1, 1.5) with $\gamma$= 0.25 and considering subgroup size as 3, 5, and 7 under LLF.

$\alpha$	*n*	Shape parameter = $\lambda$											
		0.50	1.00	1.25	1.50	1.75	2.00	2.25	2.50	2.75	3.00	4.00	5.00
		*ARL* (*SDRL*)	*ARL* (*SDRL*)	*ARL* (*SDRL*)	*ARL* (*SDRL*)	*ARL* (*SDRL*)	*ARL* (*SDRL*)	*ARL* (*SDRL*)	*ARL* (*SDRL*)	*ARL* (*SDRL*)	*ARL* (*SDRL*)	*ARL* (*SDRL*)	*ARL* (*SDRL*)
0.25	(µ, σ)	(−1.51, 2.04)	(−1.49, 0.89)	(−1.45, 0.72)	(−1.42, 0.61)	(−1.40, 0.53)	(−1.38, 0.47)	(−1.36, 0.42)	(−1.35, 0.38)	(−1.33, 0.35)	(−1.32, 0.32)	(−1.29, 0.25)	(−1.28, 0.20)
	3	2.47 (1.17)	2.75 (0.80)	2.78 (0.70)	2.83 (0.64)	2.88 (0.58)	2.92 (0.53)	2.97 (0.49)	3.00 (0.42)	3.03 (0.40)	3.05 (0.36)	3.05 (0.31)	3.01 (0.27)
	5	1.88 (0.77)	2.11 (0.47)	2.13 (0.39)	2.13 (0.36)	2.12 (0.33)	2.12 (0.32)	2.12 (0.32)	2.11 (0.31)	2.13 (0.33)	2.11 (0.32)	2.09 (0.29)	2.03 (0.19)
	7	1.60 (0.61)	1.84 (0.42)	1.90 (0.33)	1.94 (0.25)	1.97 (0.17)	1.99 (0.11)	1.99 (0.06)	1.99 (0.05)	1.99 (0.03)	1.99 (0.03)	1.99 (0.05)	1.98 (0.11)
0.50	(µ, σ)	(−0.68, 3.16)	(−0.87, 1.11)	(−0.84, 0.90)	(−0.81, 0.76)	(−0.78, 0.66)	(−0.76, 0.58)	(−0.74, 0.53)	(−0.72, 0.48)	(−0.71, 0.44)	(−0.70, 0.40)	(−0.66, 0.31)	(−0.64, 0.25)
	3	1.96 (1.01)	5.70 (2.94)	6.25 (3.06)	6.72 (3.00)	7.27 (3.10)	7.61 (3.09)	7.84 (3.01)	7.95 (2.98)	7.68 (2.81)	7.24 (2.59)	5.62 (1.82)	4.54 (1.23)
	5	1.46 (0.61)	3.97 (1.69)	4.15 (1.56)	4.34 (1.48)	4.52 (1.42)	4.55 (1.25)	4.57 (1.18)	4.44 (1.09)	4.20 (1.00)	3.88 (0.90)	3.09 (0.65)	2.65 (0.53)
	7	1.24 (0.45)	3.19 (1.17)	3.28 (1.07)	3.41 (1.00)	3.52 (0.93)	3.52 (0.80)	3.49 (0.77)	3.36 (0.71)	3.16 (0.65)	2.92 (0.62)	2.36 (0.49)	2.06 (0.25)
0.75	(µ, σ)	(0.01, 4.18)	(−0.42, 1.30)	(−0.39, 1.05)	(−0.36, 0.88)	(−0.34, 0.77)	(−0.32, 0.68)	(−0.29, 0.61)	(−0.28, 0.56)	(−0.26, 0.51)	(−0.25, 0.47)	(−0.21, 0.37)	(−0.18, 0.30)
	3	1.49 (0.69)	13.20 (10.09)	23.99 (20.21)	45.10 (39.36)	59.07 (52.32)	49.75 (42.61)	32.81 (27.26)	22.31 (16.65)	15.80 (10.53)	12.26 (7.33)	7.34 (3.14)	5.43 (1.76)
	5	1.18 (0.41)	8.48 (5.46)	15.52 (11.75)	22.41 (17.77)	23.30 (17.46)	16.24 (10.92)	10.63 (6.13)	7.90 (3.76)	6.19 (2.49)	5.20 (1.77)	3.66 (0.92)	3.02 (0.62)
	7	1.07 (0.26)	1.06 (0.25)	11.24 (7.46)	14.63 (10.05)	13.77 (8.68)	9.61 (5.19)	6.55 (2.84)	5.14 (1.87)	4.23 (1.28)	3.68 (1.00)	2.76 (0.59)	2.29 (0.46)
1.0	(µ, σ)	(0.61, 5.00)	(−0.05, 1.47)	(−0.03, 1.18)	(0.00, 1.00)	(0.03, 0.87)	(0.05, 0.77)	(0.07, 0.69)	(0.09, 0.63)	(0.11, 0.58)	(0.12, 0.54)	(0.16, 0.41)	(0.19, 0.34)
	3	1.31 (0.56)	12.59 (9.60)	62.01 (57.77)	370.68 (367.77)	342.12 (338.03)	141.43 (133.84)	66.40 (60.28)	38.90 (32.79)	26.38 (20.91)	22.65 (17.18)	8.89 (4.28)	6.40 (2.44)
	5	1.09 (0.29)	7.57 (4.99)	39.35 (35.73)	370.09 (368.89)	178.01 (172.69)	47.26 (41.65)	20.19 (14.80)	12.40 (7.76)	8.85 (4.72)	7.15 (3.18)	4.15 (1.19)	3.37 (0.77)
	7	1.02 (0.15)	5.58 (3.17)	28.08 (24.05)	370.89 (366.23)	103.09 (96.58)	24.88 (19.76)	11.43 (6.92)	7.46 (3.57)	5.69 (2.26)	4.75 (1.61)	3.05 (0.71)	2.56 (0.55)
1.25	(µ, σ)	(1.17, 5.69)	(0.28, 1.65)	(0.29, 1.31)	(0.32, 1.11)	(0.35, 0.96)	(0.37, 0.86)	(0.39, 0.77)	(0.41, 0.70)	(0.43, 0.64)	(0.45, 0.60)	(0.49, 0.46)	(0.52, 0.38)
	3	1.32 (0.56)	7.08 (4.74)	17.28 (13.83)	31.99 (27.51)	40.31 (35.12)	35.71 (30.64)	42.15 (36.38)	36.27 (29.78)	28.51 (22.04)	23.20 (17.39)	11.22 (6.17)	7.63 (3.39)
	5	1.04 (0.21)	4.58 (2.49)	10.78 (7.52)	20.34 (16.36)	22.16 (17.43)	21.14 (16.01)	17.36 (12.27)	13.67 (8.39)	10.44 (5.59)	8.70 (4.17)	4.93 (1.60)	3.77 (0.98)
	7	1.01 (0.10)	3.59 (1.71)	8.08 (5.08)	14.32 (10.43)	15.03 (10.69)	13.55 (8.81)	11.07 (6.33)	8.55 (4.09)	6.75 (2.75)	5.71 (2.06)	3.54 (0.92)	2.83 (0.62)

$\alpha$	*N*	Shape parameter = $\lambda$											
		0.50	1.00	1.25	1.50	1.75	2.00	2.25	2.50	2.75	3.00	4.00	5.00
		*ARL* (*SDRL*)	*ARL* (*SDRL*)	*ARL* (*SDRL*)	*ARL* (*SDRL*)	*ARL* (*SDRL*)	*ARL* (*SDRL*)	*ARL* (*SDRL*)	*ARL* (*SDRL*)	*ARL* (*SDRL*)	*ARL* (*SDRL*)	*ARL* (*SDRL*)	*ARL* (*SDRL*)
1.50	(µ, σ)	(1.67, 6.26)	(0.58, 1.86)	(0.58, 1.44)	(0.61, 1.21)	(0.64, 1.05)	(0.66, 0.94)	(0.68, 0.84)	(0.70, 0.77)	(0.72, 0.71)	(0.74, 0.65)	(0.79, 0.51)	(0.82, 0.41)
	3	1.15 (0.39)	4.44 ( 2.58)	7.41 (4.76)	9.51 (6.17)	9.74 (6.22)	9.69 (5.88)	9.36 (5.20)	9.04 (4.82)	8.59 (4.23)	8.17 (3.69)	6.86 (2.36)	6.01 ( 1.67)
	5	1.06 ( 0.24)	3.08 ( 1.48)	5.16 ( 2.83)	6.34 ( 3.55)	6.24 ( 3.27)	6.04 ( 2.88)	5.75 ( 2.54)	5.52 ( 2.23)	5.17 ( 1.85)	4.93 ( 1.66)	4.33 ( 1.17)	3.62 ( 0.73)
	7	1.00 ( 0.08)	4.39 ( 2.15)	4.04 ( 1.92)	4.95 ( 2.47)	4.79 ( 2.18)	4.58 ( 1.89)	4.86 ( 1.91)	4.19 ( 1.41)	3.98 ( 1.21)	3.77 ( 1.01)	3.24 ( 0.65)	2.35 ( 0.48)
2.00	(µ, σ)	(2.57, 7.16)	(1.16, 2.43)	(1.11, 1.70)	(1.13, 1.41)	(1.15, 1.23)	(1.18, 1.09)	(1.20, 0.98)	(1.23, 0.89)	(1.25, 0.82)	(1.26, 0.76)	(1.32, 0.59)	(1.36, 0.48)
	3	1.10 ( 0.31)	2.44 (1.25)	3.50 (1.79)	3.84 (1.84)	3.81 (1.69)	2.26 (0.65)	3.61 (1.34)	3.49 (1.17)	3.39 (1.09)	3.34 ( 0.96)	3.11 (0.70)	2.98 (0.55)
	5	1.01 ( 0.12)	1.79 ( 0.77)	2.58 ( 1.09)	2.81 ( 1.12)	2.80 ( 1.05)	2.72 ( 0.91)	2.63 ( 0.79)	2.55 ( 0.69)	2.49 ( 0.63)	2.45 ( 0.58)	2.09 ( 0.29)	2.15 ( 0.36)
	7	1.00 ( 0.04)	1.52 ( 0.60)	2.18 ( 0.83)	2.06 ( 0.71)	2.34 ( 0.76)	2.26 ( 0.66)	2.21 ( 0.58)	2.14 ( 0.49)	2.10 ( 0.43)	2.08 ( 0.39)	2.00 ( 0.22)	1.99 ( 0.11)
2.50	(µ, σ)	(3.33, 7.82)	(1.74, 3.18)	(1.59, 2.05)	(1.59, 1.61)	(1.62, 1.39)	(1.64, 1.23)	(1.67, 1.11)	(1.69, 1.01)	(1.72, 0.93)	(1.73, 0.86)	(1.80, 0.67)	(1.84, 0.55)
	3	1.05 (0.23)	1.68 (0.80)	2.35 (1.08)	2.58 (1.10)	2.57 (0.96)	2.52 (0.88)	2.44 (0.76)	2.40 (0.69)	2.34 (0.61)	2.32 (0.56)	2.18 (0.42)	2.10 ( 0.31)
	5	1.00 ( 0.07)	1.32 ( 0.52)	2.04 (1.09)	1.99 ( 0.75)	1.98 ( 0.67)	1.94 ( 0.60)	1.91 ( 0.54)	1.89 ( 0.49)	1.91 ( 0.31)	1.86 ( 0.41)	1.85 ( 0.35)	1.86 ( 0.34)
	7	1.00 ( 0.03)	1.15 ( 0.36)	1.55 ( 0.60)	1.68 (0.61)	1.66 ( 0.56)	1.66 ( 0.53)	1.62 ( 0.51)	1.60 ( 0.50)	1.57 ( 0.49)	1.57 ( 0.49)	1.47 ( 0.49)	1.38 ( 0.48)
3.00	(µ, σ)	(4.00, 8.33)	(2.35, 4.00)	(2.08, 2.58)	(2.03, 1.86)	(2.04, 1.55)	(2.07, 1.37)	(2.10, 1.24)	(2.12, 1.13)	(2.15, 1.04)	(2.17, 0.96)	(2.24, 0.75)	(2.29, 0.61)
	3	1.05 (0.23)	1.36 (0.58)	1.78 (0.82)	2.01 (0.83)	2.01 (0.75)	2.00 ( 0.67)	1.95 (0.61)	1.94 (0.55)	1.91 (0.50)	1.90 (0.44)	1.88 (0.35)	1.89 (0.31)
	5	1.00 ( 0.09)	1.12 ( 0.34)	1.39 ( 0.55)	1.57 ( 0.60)	1.58 ( 0.58)	1.55 ( 0.54)	1.52 ( 0.52)	1.50 ( 0.51)	1.46 ( 0.50)	1.48 ( 0.56)	1.45 ( 0.54)	1.26 ( 0.44)
	7	1.00 ( 0.02)	1.03 ( 0.19)	1.21 ( 0.42)	1.41 ( 0.53)	1.59 ( 0.62)	1.67 ( 0.65)	1.72 ( 0.67)	1.21 ( 0.41)	1.17 ( 0.38)	1.14 ( 0.35)	1.05 ( 0.23)	1.01 ( 0.11)
4.00	(µ, σ)	(5.12, 9.07)	(3.61, 5.53)	(3.15, 3.99)	(2.93, 2.81)	(2.86, 2.07)	(2.86, 1.68)	(2.88, 1.48)	(2.91, 1.35)	(2.94, 1.24)	(2.96, 1.15)	(3.04, 0.89)	(3.10, 0.73)
	3	1.04 (0.20)	1.13 (0.36)	1.27 ( 0.51)	1.41 (0.59)	1.48 (0.59)	1.46 (0.55)	1.44 (0.52)	1.41 (0.50)	1.39 (0.49)	1.36 (0.48)	1.27 (0.44)	1.18 (0.38)
	5	1.00 ( 0.06)	1.02 ( 0.16)	1.07 ( 0.27)	1.17 ( 0.38)	1.19 ( 0.40)	1.15 ( 0.36)	1.12 ( 0.33)	1.09 ( 0.29)	1.07 ( 0.25)	1.05 ( 0.22)	1.01 ( 0.10)	1.00 ( 0.04)
	7	1.00 ( 0.01)	1.00 ( 0.06)	1.02 ( 0.14)	1.06 ( 0.25)	1.07 ( 0.26)	1.04 ( 0.21)	1.02 ( 0.16)	1.01 ( 0.11)	1.00 ( 0.08)	1.00 ( 0.08)	1.00 ( 0.01)	1.0 (0.0)

Step 1: Establishing the control limits

i. To commence, establish the initial control limits by computing the values for UCL and λ.
ii. Generate a random sample of size n to depict the in-control process, utilizing normal distributions.
iii. Calculate the statistic required for the suggested control chart.

Verify whether the plotted statistic lies within the UCL; if so, proceed to steps (iii–iv) once more.

Step 2: Assessing the out-of-control average run length (ARL)

i. Generate a random sample reflecting a shifted process.
ii. Calculate the statistic required for the suggested control chart.
iii. Should the plotted statistic fall within the UCL, iterate through steps (i–ii). Otherwise, document the count of generated points, signifying a single out-of-control run length.
iv. Iterate through the aforementioned process (i–iii) 50, 000 times to ascertain the out-of-control ARL_1_ and SDRL_1_.

## Main findings

The main findings of the current study are given below:

- The effectiveness of the proposed Max-EWMA CC using the Weibull process for simultaneously monitoring both process mean and variance, especially in identifying subtle to moderate shifts, becomes apparent when analyzing the run length profiles provided in all four tables related to this CC. These profiles showcase how the CC performs over a range of scenarios, and the consistent trend across these tables indicates that the CC is adept at promptly detecting deviations in both process mean and variance, making it a valuable tool for maintaining process quality and consistency.
- The simulation results clearly demonstrate that the performance of the proposed Bayesian CC for simultaneous monitoring of processes improves as the smoothing constant decreases. In other words, when the smoothing constant is reduced, the CC becomes more sensitive and effective at promptly identifying shifts in the process mean and variance. This finding implies that choosing a lower smoothing constant may, in some cases or applications, improve process quality and reliability by facilitating better monitoring and faster identification of deviations from the expected process parameters.
- The variation in sample size is one of the important factors that we have carefully examined in the context of our study. Our analysis's findings provide an important and persuasive insight. It is evident that there is a notable and significant improvement in the efficiency and performance of the proposed Bayesian Max-EWMA CC with increasing sample size. Put practically, this means that the CC can detect changes in process mean and variance more quickly and accurately when larger sample sizes are used. This improvement is particularly important because it can lead to more reliable and robust process monitoring, contributing to better overall process quality and consistency.

## Real data application

Many researchers commonly employ the practice of demonstrating the functionality and effectiveness of proposed CCs using actual datasets and simulated scenarios. In this context, we analyze a real-life dataset to showcase the capabilities of the proposed CC. Monitoring the tensile strength of fibrous composites is crucial in industries ensuring material safety for aerospace and bridge construction. To achieve this, we examine the real-life dataset referenced in ^26^, which specifically outlines the breaking strengths of carbon fibers used in manufacturing these composite materials, as detailed in Table 5. These insights stem from research conducted at the U.S. Army Materials Technology Laboratory in Watertown, Massachusetts. The dataset consists of 20 samples, each comprising a sample size of n = 5, following the Weibull distribution with a scale parameter (η = 2.9437) and shape parameter (θ = 2.7929). Initially, 15 random samples of size n = 5 are drawn without replacement and marked as in-control samples. Subsequently, the dataset is altered by adding 1 to each observation. Following this modification, 10 random samples of size n = 5 are drawn without replacement and regarded as out-of-control samples. Both charts are employed to monitor variations in the process mean, and the resulting computations are presented in Table 6.

**Table 5:** Data set related to breaking strengths of carbon fibers.

Sample number	Breaking stresses (GPa) of carbon fibers					Transformed standard normal from Weibull				
1	4.91	2.85	2.12	5.08	2.76	2.1603	0.2502	−0.4407	2.3212	0.1665
2	3.68	3.15	1.84	2.97	2.95	1.0159	0.5277	−0.7190	0.3614	0.3429
3	3.11	2.95	4.20	3.19	1.87	0.4908	0.3429	1.4964	0.5646	−0.6886
4	3.56	5.56	1.17	2.93	1.22	0.9054	2.7800	−1.4527	0.3243	−1.3927
5	3.31	4.38	3.15	3.33	1.59	0.6752	1.6636	0.5277	0.6936	−0.9785
6	2.83	2.55	3.11	1.73	1.18	0.2316	−0.0297	0.4908	−0.8316	−1.4406
7	2.50	2.79	2.17	2.48	3.19	−0.0767	0.1944	−0.3921	−0.0956	0.5646
8	2.38	1.08	1.18	3.19	0.81	−0.1904	−1.5638	−1.4406	0.5646	−1.9297
9	2.82	0.39	1.41	2.43	2.73	0.2223	−2.6946	−1.1744	−0.1429	0.1386
10	2.35	0.81	3.19	5.08	4.70	−0.2189	−1.9297	0.5646	2.3212	1.9627
11	1.57	3.39	2.03	2.48	2.81	−0.9998	0.7489	−0.5290	−0.0956	0.2130
12	2.97	2.03	2.79	3.15	3.75	0.3614	−0.5290	0.1944	0.5277	1.0804
13	3.56	2.00	1.89	2.76	2.79	0.9054	−0.5587	−0.6685	0.1665	0.1944
14	3.31	2.48	3.28	0.39	3.15	0.6752	−0.0956	0.6476	−2.6946	0.5277
15	3.51	0.81	2.81	0.85	2.95	0.8594	−1.9297	0.2130	−1.8716	0.3429
16	2.59	3.03	3.48	5.42	4.11	0.0078	0.4169	0.8317	2.6454	1.4130
17	4.39	4.33	3.05	4.68	4.60	1.6730	1.6171	0.4354	1.9439	1.8690
18	3.81	4.09	2.57	2.25	1.98	1.1358	1.3945	−0.0109	−0.3147	−0.5785
19	3.00	3.38	2.36	4.22	4.65	0.3891	0.7397	−0.2094	1.5149	1.9158
20	3.76	3.88	3.95	2.89	3.83	1.0897	1.2003	1.2650	0.2873	1.1542
21	4.60	4.39	4.68	5.20	3.59	1.8690	1.6730	1.9439	2.4352	0.9330
22	2.92	3.59	2.69	5.20	2.89	0.3151	0.9330	0.1013	2.4352	0.2873
23	4.75	1.98	2.92	4.31	3.87	2.0097	−0.5785	0.3151	1.5985	1.1911
24	2.80	4.33	4.56	3.96	3.03	0.2037	1.6171	1.8316	1.2742	0.4169
25	6.56	3.82	2.18	3.95	2.84	3.7604	1.1450	−0.3824	1.2650	0.2409

**Table 6:** The values and out of control status proposed Bayesian Max-EWMA under SELF and LLF, with $\lambda$ = 0.10.

Sample #	Proposed Bayesian Max-EWMA under SELF	UCL	Out-of-control status	Proposed Bayesian Max-EWMA under LLF	UCL	Out-of-control status
1	0.1153	1.6654	0	0.1225	1.6521	0
2	0.1678	1.6654	0	0.2649	1.6521	0
3	0.2209	1.6654	0	0.5425	1.6521	0
4	0.3492	1.6654	0	0.5793	1.6521	0
5	0.4164	1.6654	0	0.7055	1.6521	0
6	0.4359	1.6654	0	0.8701	1.6521	0
7	0.6002	1.6654	0	0.7589	1.6521	0
8	0.6508	1.6654	0	0.8891	1.6521	0
9	0.7089	1.6654	0	0.8864	1.6521	0
10	0.7512	1.6654	0	0.8711	1.6521	0
11	0.8653	1.6654	0	1.0055	1.6521	0
12	1.0164	1.6654	0	1.0835	1.6521	0
13	1.1442	1.6654	0	1.1308	1.6521	0
14	1.1255	1.6654	0	1.2065	1.6521	0
15	1.1835	1.6654	0	1.2176	1.6521	0
16	1.1794	1.6654	0	1.2005	1.6521	0
17	1.2055	1.6654	0	1.3013	1.6521	0
18	1.3781	1.6654	0	1.3895	1.6521	0
19	1.5150	1.6654	0	1.4339	1.6521	0
20	1.5070	1.6654	0	1.6128	1.6521	0
21	1.4995	1.6654	0	1.7285	1.6521	1
22	1.6310	1.6654	0	1.7148	1.6521	1
23	1.7426	1.6654	1	1.8754	1.6521	1
24	1.7993	1.6654	1	1.8448	1.6521	1
25	1.7791	1.6654	1	1.8092	1.6521	1

**Figure 1:**
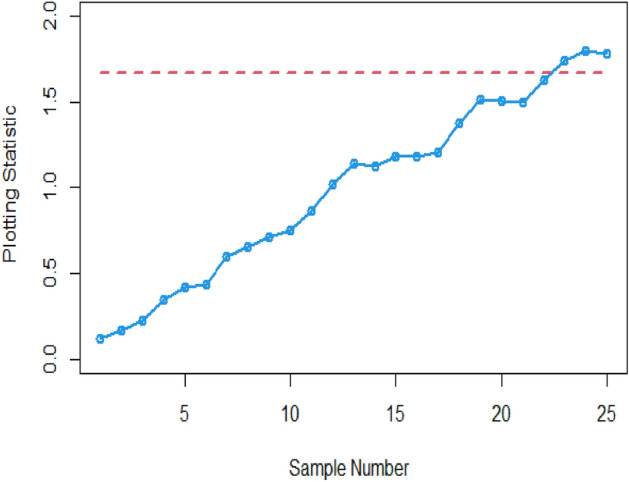
Using SELF, the Bayesian Max-EWMA CC using Weibull process for jointly monitoring with $\lambda = 0.10$.

**Figure 2:**
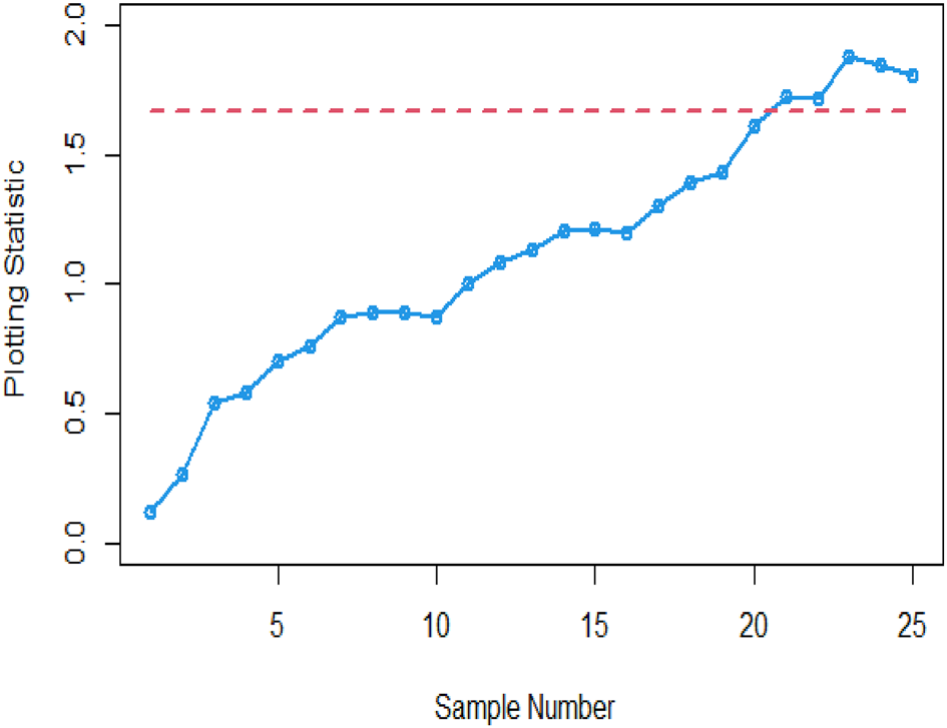
Using LLF, Bayesian Max-EWMA CC for using Weibull process for jointly monitoring with $\lambda = 0.10$.

Figures 1 and 2 provide a visual representation of the implementation of the provided Bayesian Max-EWMA CC, designed for the simultaneous monitoring of both process mean and dispersion. This monitoring employs both the SELF and LLF approaches. A thorough examination of these charts reveals clear signals indicating that the process has gone out of control in the 23rd and 21st samples, especially when considering a smoothing constant value of 0.10. The identified departure from the normal process state, resulting in an out-of-control scenario, can be ascribed to two main factors: alterations in either the process mean or variance. These alterations stem from modifications in the shape and scale parameters of the Weibull distribution, thereby influencing the distribution's properties and giving rise to the observed deviations.

## Conclusion

In this study, we introduce an innovative Bayesian Max-EWMA CC using the Weibull process designed for the simultaneous monitoring of the process mean and variance. This CC incorporates informative prior distributions and integrates two distinct LFs within the context of posterior distribution. The performance of this novel approach was rigorously evaluated through a comprehensive analysis, with results presented in Tables 1, 2, 3 and 4. These assessments employ crucial metrics such as ARL and SDRL. We conduct a practical case study focused on the hard bake process in semiconductor manufacturing. Interestingly, the proposed Bayesian Max-EWMA CC shows excellent performance in identifying out-of-control signals in the process when applied to posterior distributions. Crucially, the knowledge acquired from this research could be applied to the creation of other memory-type CCs, improving their efficacy in a variety of industrial applications. Extending this novel method to different kinds of CC instead of just nonnormal distributions can lead to a more thorough comprehension of the underlying data patterns. This broader application enables the early detection of potential quality issues in different domains and allows for swift corrective actions, thereby reducing the risk of costly errors and defects. This method is essential for quickly spotting irregularities in patient data, enabling prompt interventions, and enhancing the quality of patient care in real-world situations like healthcare. In manufacturing, extending this approach to non-normal distributions and diverse CC types aids in identifying variations in the production process, ultimately leading to improved product quality and a reduction in waste.

## Data Availability

The statement signifies that the datasets used or analyzed in the study are not publicly available but can be acquired from the corresponding author upon a reasonable request. It suggests the author holds the data and is open to sharing it with interested parties in an appropriate manner.
